# Association between Self-Reported Childhood Difficulties and Obesity and Health-Related Behaviors in Adulthood—A Cross-Sectional Study among 28,047 Adults from the General Population

**DOI:** 10.3390/ijerph19031395

**Published:** 2022-01-26

**Authors:** Tonje Holte Stea, Herolinda Shatri, Siri Håvås Haugland, Annette Løvheim Kleppang

**Affiliations:** 1 Department of Health and Nursing Science, University of Agder, 4604 Kristiansand, Norway; herolinda.shatri@gmail.com (H.S.); annette.kleppang@uia.no (A.L.K.); 2 Department of Child and Adolescence Mental Health, Sørlandet Hospital, 4604 Kristiansand, Norway; 3 Department of Psychosocial Health, University of Agder, 4876 Grimstad, Norway; siri.h.haugland@uia.no

**Keywords:** childhood difficulties, obesity, diet, leisure-time physical activity, substance use, adults

## Abstract

The aim of the present study was to examine the associations between self-reported childhood difficulties, weight status, and lifestyle behaviors among a representative sample of Norwegian adults. This cross-sectional study included 28,047 adults (>18 years old) living in southern Norway. A self-report questionnaire was used to assess information about the overall quality of the respondents’ childhood retrospectively in addition to current weight status and current lifestyle behaviors. Multivariable logistic regression models adjusted for gender, age, and educational level showed that evaluating childhood as difficult was associated with increased odds of obesity (OR: 1.29; 95% CI; 1.16–1.44) in adulthood. Moreover, a difficult childhood was associated with increased odds of unhealthy lifestyle behaviors in adulthood, including low consumption of fruit and berries (1.21; 1.09–1.34) and fish (1.43; 1.30–1.57), high consumption of sugar-sweetened beverages (1.30; 1.14–1.48), low level of physical activity (1.10; 1.01–1.21), smoking cigarettes (1.78; 1.61–1.97), and using smokeless tobacco (1.20; 1.07–1.36). Overall, results from the present study suggest that experiencing childhood as difficult is associated with an increased risk of obesity and a range of unhealthy lifestyle behaviors in adulthood. Thus, our findings highlight the importance of identifying and providing support to children in difficult life circumstances in addition to customized and targeted public health efforts in adulthood.

## 1. Introduction

Obesity is global, complex, multifactorial disease with personal, environmental, genetic, and social influences [1]. As obesity rates continue to accelerate worldwide, increased knowledge about the multiple contributing factors to obesity has been warranted in order to improve obesity prevention and treatment [2,3].

Some studies have indicated an association between adverse childhood experiences (ACEs), which involves potentially traumatic events, such as exposure to abuse, neglect, or household dysfunction, and prevalence of obesity [4,5], whereas other studies have reported only modest or no associations [6,7,8]. A study among Norwegian adults reported a relatively strong, graded relationship between experiencing childhood difficulties and health risk indicators, such as increased resting heart rate, waist circumference, waist-hip ratio, and body mass index (BMI) [9]. Studies have also confirmed that ACEs elevates the risk of psychiatric problems and chronic disease over a lifetime, and with increasing childhood difficulties, the prevalence of most eligible diseases and disorders increase in a dose-response manner [10,11,12]. Moreover, ACEs impose a substantial financial burden to society: in Europe, the total annual costs attributable to ACEs have been estimated to USD 581 billion, and a modest 10% reduction in prevalence of ACEs has suggested to be equivalent to annual savings of 3 million disability-adjusted life years (DALYs) [13].

Some studies have suggested that obesity-related behaviors are more common among individuals with ACEs, and this may partly explain the relationship between ACE and health problems [9,14]. Few studies have investigated whether childhood difficulties may contribute to an individual’s food choices, but some results have indicated an inverse association between exposure to ACEs and consumption of fruit and vegetables [15,16,17]. A review study aiming to identify plausible mechanisms explaining the role of ACEs in cardiovascular disease risk reported mixed results of studies examining the relationship between ACEs and level of physical activity in adults [18]. Although studies among U.S. adults have shown that ACEs were associated with increased likelihood of being less physically active [11,19], another study among nationally representative UK adults did not show any relationship between ACEs and level of physical activity [20]. On the other hand, several studies have confirmed a positive association between ACEs, risk of smoking, excessive alcohol use, and drug abuse [10,14,21,22,23].

It has been well established that adverse experiences in childhood are not randomly distributed across population groups and that socioeconomic disadvantage and female gender has been identified as strong risk factors for ACEs [9,24,25,26,27]. Moreover, ACEs are associated with a substantially increased risk of low educational achievement and unemployment [28], which also have been linked to unhealthy lifestyle behaviors [29].

Identifying lifestyle behaviors and health indicators associated with a difficult childhood may contribute to developing tailored health-promoting interventions targeting vulnerable groups. Traditionally, ACEs have been measured by mapping the occurrence of specific types of adverse childhood experiences, focusing on quantifying experiences. However, a less common approach is to ask more subjective, global questions about childhood quality, facilitating shorter questionnaires more suitable for larger surveys. Global questions may also be less intrusive to the respondents compared to questions of very specific and often traumatic adverse childhood experiences. Several studies have found a relationship between the subjective evaluation of childhood and health-related outcomes [30,31], but to our knowledge, this approach has not been used related to obesity and obesity-related behaviors. Thus, the present study’s main aim was to examine whether self-reported childhood difficulties were associated with increased prevalence of obesity and unhealthy lifestyle behaviors among a representative sample of Norwegian adults.

## 2. Materials and Methods

### 2.1. Study Design and Population

The present cross-sectional study was part of the Norwegian Counties Public Health Survey, designed to collect information about health-related behaviors, health, well-being, and quality of life among the adult population across Norway. A random sample of 75,191 adults (>18 years of age) living in the southern region of Norway was drawn from the National Register (31.6% of the adult population in this region). After the removal of institutionalized and deceased individuals and those registered in the Contact and Reservation Register with unverified contact information or an address registered outside the included municipalities, a total of 61,611 adults (25.9% of the region population) were invited by SMS and e-mail to participate in the study between September and October 2019. To disseminate information and recruit participants, written and oral information about the study was broadcasted through social media (Facebook, local webpages for the county and municipalities), regional and local newspapers, and television. To further increase the participation rate, gift cards worth NOK 4000 (EUR 380) were distributed to six random participants. All participants received written information about the study according to the standards of the Norwegian Data Inspectorate and consented online by participating in the study. A total of 28,047 residents, 14,925 females (53.2%) and 13,122 males (46.8%), agreed to participate (response rate 45.5%). Approximately 15 min was used to complete the online questionnaire, and participants had the opportunity to withdraw from the study at any time and have all personal information erased. The National Institute of Public Health was responsible for collecting and anonymizing data analyzed by independent researchers who did not participate in the data collection process or had access to personally identifiable information. Furthermore, the Norwegian Institute of Public Health was legally responsible for the Norwegian Counties Public Health Survey, and the study was conducted in line with the Declaration of Helsinki. Ethical approval and research clearance were obtained from the ethical committee of the University of Agder and the Regional Committee for Medical Research Ethics (file number 162353/2020).

### 2.2. Measures

All measures were based on self-reported data. Questions, response alternatives, and variable definitions are presented in Table 1.

To identify individuals that experienced childhood as difficult, the present study used a single question that refers to the participant’s subjective, global perception of her/his childhood and has proven to be strongly related to negative health outcomes [9]. The specific question that was used to assess childhood difficulties in the present study was originally developed for the Trøndelag Health Study (HUNT) and has shown a graded relationship with adult multimorbidity, individual disease burden, and biological perturbations [32]. Use of this question to assess childhood difficulties has also been recommended by a feasibility study among patients living with binge eating disorder and comorbid obesity [33]. The phrasing of the question was developed with respect to the local linguistic and cultural context, supported by a linguist, and a study by Vederhus and coworkers [31] has indicated that the question would be an empirically supported component when assessing adverse childhood experiences.

Body mass index (BMI) was calculated based on respondent’s information on height in cm and weight in kg without clothes and shoes on (kg/m^2^). Pregnant females had to state their weight pre-pregnancy. BMI was calculated to identify underweight (<18.5), normal weight (18.5–24.9), overweight (25–29.9), obesity class I (30–34.9), and obesity class II + (≥35). Further, BMI was dichotomized into living with obesity (≥30) and not living with obesity (<30).

Diet and beverage consumption were assessed by asking respondents four questions on how often they eat fruit and berries, vegetables, and fish and how often they drink sugar-sweetened beverages. The response alternatives were dichotomized into having high or low consumption of the selected food items and beverages using cut-off values that have previously been used in similar studies among adults [34,35].

Leisure-time physical activity was assessed using three questions reflecting frequency, intensity, and duration. To estimate whether participants were active according to national authorities and WHO recommendations [36], the frequency question was coded as follows: never (coded: 0), <once/week (0.5), 2–3 times/week (2.5), 4–5 times a week (4.5), and ≥6 times/week (6.5). Further, the question regarding physical activity duration was coded as follows: <15 min (coded: 8), 15–29 min (22), 30–60 min (45), and >60 min (75). Further, data reflecting frequency, intensity, and duration of leisure-time physical activity were combined to identify individuals who achieved the recommended levels of physical activity of at least 150 min of moderate-intensity aerobic physical activity or at least 75 min of vigorous-intensity aerobic physical activity.

Information about alcohol use was assessed by asking respondents whether they had ever consumed alcohol or not. Response alternatives were “yes” and “no.” For responders who answered “yes,” the Alcohol-Use Disorders Identification Test Consumption (AUDIT-C) was used to assess alcohol habits and identify potential alcohol problems. AUDIT-C is a widely used screening instrument covering the amount and frequency of a person’s alcohol use using three questions [37]. A total sum score between 0 and 12 points was calculated, and in line with recommendations based on previous studies, we used a cut-off score of >4 for women and >5 for men to indicate excessive alcohol use [38,39,40,41,42].

Smoking cigarettes and the use of smokeless tobacco were assessed using a single question about how often participants smoke/use smokeless tobacco. In line with previous studies, results were dichotomized to identify current status on smoking habits and use of smokeless tobacco [15].

### 2.3. Control Variables

Information about age and gender was retrieved from the Central Population Register, whereas information about the highest completed educational level was assessed by the online questionnaire. Age was used as a continuous variable.

### 2.4. Statistical Analysis

Descriptive analyses were stratified according to childhood experiences. Categorical variables were expressed as frequencies and percentages whereas continuous variables were presented as means and standard deviations. Pearson chi-square tests were used to identify differences in categorical variables, including BMI status; consumption of fruit and berries, vegetables, fish, and sugar-sweetened beverages; physical activity; risk drinking; and smoking and use of smokeless tobacco, according to childhood experiences. Age was presented as a continuous variable in the present study, and differences in age according to childhood experiences were tested using ANOVA test. Logistic regression analyses were applied to investigate possible associations between self-perceived childhood difficulties and prevalence of obesity, dietary habits, leisure-time physical activity, and substance use. All models were adjusted for gender, age, and educational level. Results were reported as odds ratio (OR), with 95% confidence interval (CI). Pearson correlation tests revealed low pairwise correlations between independent variables in the models, indicating that multicollinearity was not present. SPSS statistical program (version 25) was used for all analyses, and the level of statistical significance was set to *p* < 0.05.

## 3. Results

Table 2 presents background characteristics and risk health behavior of participants stratified according to participants’ evaluation of childhood quality. Distributed by reported childhood experiences, 13,938 participants (49.8%) reported childhood as being very good, 8252 (29.5%) good, 3460 (12.2%) average, 1657 (5.9%) difficult, and 689 (2.5%) very difficult. In general, individuals reporting childhood as difficult or very difficult were more often female, younger age, and with lower educational level. Further, a significant trend was observed from very good to very difficult childhood indicating that among those who evaluated childhood as difficult a higher proportion reported to live with underweight and obesity and to have low consumption of fruit and berries and fish, high consumption of sugar-sweetened beverages, and low level of physical activity. A significant trend was also observed between childhood experiences, smoking cigarettes, and using smokeless tobacco but not between childhood experiences and excessive alcohol use.

Table 3 and Table 4 show that having a difficult childhood was associated with increased odds of being overweight or obese (OR 1.29, 95% CI; 1.16–1.44), low consumption of fruit and berries (1.21; 1.09–1.34), fish (1.43; 1.30–1.57), and high consumption of sugar-sweetened beverages (1.30; 1.14–1.48). Further, describing childhood as difficult was associated with increased odds of low level of physical activity (1.10; 1.01–1.21) and being current smokers (1.78; 1.61–1.97) and users of smokeless tobacco (1.20; 1.07–1.36) compared to those reporting no childhood difficulties (reference group). Results from logistic regression analyses did not show an association between having a difficult childhood and low consumption of vegetables and excessive alcohol use.

Control variables indicated that men have increased odds of living with obesity (1.18; 1.00–1.25), having low consumption of fruit and berries (2.37; 2.24–2.51) and fish (2.40; 2.27–2.52), high consumption of sugar-sweetened beverages (1.07; 1.01–1.14), low level of physical activity (1.98; 1.82–2.15), and being a current smoker (1.07; 1.00–1.15) and user of smokeless tobacco (2.59; 2.41–2.80) compared to women (reference group). Control variables also indicated that older adults had increased odds of living with obesity (1.00; 1.00–1.01) and decreased odds of low consumption of fruit and berries (0.97; 0.97–0.97) and vegetables (0.98; 0.98–0.99) and fish (0.96; 0.96–0.97), high consumption of sugar-sweetened beverages (0.97; 0.96–0.97), excess alcohol drinking (0.97; 0.97–0.98), and being a current smoker (1.00;1.99–1.00) and user of smokeless tobacco (0.95;0.95–0.95) compared to younger adults. Finally, university or college education was associated with decreased odds of living with obesity (0.56; 0.51–0.62), having low consumption of fruit and berries (0.61; 0.56–0.67) and vegetables (0.54; 0.50–0.59) and fish (0.50; 0.45–0.54), and high consumption of sugar-sweetened beverages (0.35; 0.31–0.40), low level of physical activity (0.66; 0.61–0.72), and being a current smoker (0.65; 0.59–0.72) and user of smokeless tobacco (0.84; 0.74–0.94) compared to those having no more than primary education (reference group).

## 4. Discussion

Results from the present study confirmed that self-perceived childhood difficulties were associated with increased odds of living with obesity and being involved in risk behaviors, such as having unhealthy dietary habits, low level of physical activity, smoking cigarettes, and use of smokeless tobacco, in a large sample of Norwegian adults. These findings are consistent with an increasing body of evidence linking childhood difficulties to poorer health and adverse lifestyle behaviors in a life-course perspective [9,43,44].

In line with results from our study, previous studies have also confirmed an association between ACEs and living with obesity [45,46,47,48,49,50]. Moreover, a meta-analysis of cross-sectional studies reported 46% increased odds of adult obesity following exposure to multiple ACEs [51]. Suggested mechanisms for the association between ACEs and obesity in adulthood include biological, psychological, and environmental factors [52], and the most commonly cited explanation linking ACEs to adult obesity are social disruption, health behaviors, and chronic stress response [51]. A shift toward hypothalamic-pituitary-adrenal (HPA) hypoactivity has been reported among individuals with ACEs, which has been proposed to contribute towards altered cortisol secretion, chronic low-grade inflammation, and dysregulated hemodynamic and autonomic function [53]. A scoping review, which also reported that biomarkers of ACEs related to inflammation, cardio/metabolic systems, genetics, and endocrine systems had been identified in adulthood, concluded that health behaviors, emotional distress, social relationships, and socioeconomic factors may partly explain some of these associations [54]. 

Specifically, our results showed that evaluating childhood as difficult was associated with low consumption of fruit and berries and fish and high consumption of sugar-sweetened beverages. On the other hand, no association was shown between childhood difficulties and consumption of vegetables. Although few previous studies have examined the association between having a difficult childhood and dietary behaviors in adulthood, a longitudinal study indicated that exposure to ACEs predicted a decreased consumption of fruit and vegetables [17]. Furthermore, a cross-sectional study confirmed that participants with ACEs had increased likelihood of having low daily fruit and vegetable consumption and preference for “feel good” foods associated with increased risk of obesity [16]. Other studies have confirmed increased adherence to unhealthy dietary behaviors characterized by low consumption of natural foods and high consumption of processed and obesogenic foods among individuals with ACEs [55,56]. Individuals reporting ACEs may also eat or refrain from eating as a form of coping with their childhood traumas, and this pattern may continue into adulthood [45,46]. This pathway has been confirmed by studies showing a significant association between prevalence of obesity, post-traumatic stress disorder (PTSD), and depressive symptoms [57,58]. A possible explanation for the association is that ACEs may cause structural and functional changes to the brain that may have a negative, long-lasting effect on executive function [59]; in return, executive dysfunction may result in a less healthy lifestyle and influence, for example, choosing immediate rewards (e.g., energy dense food) instead of large, delayed rewards (e.g., health) [60].

Moreover, results from our study showed that self-perceived childhood difficulties were associated with low levels of physical activity in adulthood. According to a review study, results from previous studies investigating the relationship between exposure to ACE and physical activity in adulthood have been mixed [18]. A nationally representative survey among adult residents in the UK found no association between history of ACE and level of physical activity [20], whereas a systematic review and meta-analysis reported a weak or modest association between ACE history and physical inactivity [7]. Other studies among European adults have shown that individuals reporting ACEs had lower levels of physical activity compared to those with no ACEs [9,61]. These inconsistent results may indicate that some individuals with ACE history, especially those who have experienced physical and sexual abuse, use physical activity as a coping mechanism [18]. In line with this hypothesis, a recent study found that engaging in regular physical activity may protect functional independence from the detrimental effects of ACEs and depression [62].

Similar to our results, a previous cross-sectional study found limited evidence supporting an association between ACEs and problem alcohol use [42]. Another study, however, showed that exposure to ACE was associated with a two- to four-fold increase in excessive alcohol consumption [43]. Despite mixed results, some researchers have argued that alcohol consumption seems to be a common method of self-medication among adults [63], and others have suggested that individuals also might use excessive alcohol drinking as a coping mechanism to escape, avoid, or regulate unpleasant emotions [64,65]. Nevertheless, this hypothesis was not supported by our findings.

In line with results from the present study, a few previously published studies have confirmed an association between exposure to ACEs and smoking cigarettes in adulthood [17,22,24,66,67]. In addition, our results indicated an association between exposure to ACEs and increased odds of using smokeless tobacco. A retrospective cohort study showed that 70% of the adult respondents with ACE reported a history of parental smoking and that exposure to adult smoking and tobacco use in childhood may most likely also affect smoking habits [68] and use of smokeless tobacco in adulthood [67]. As nicotine has demonstrable psychoactive benefits in affect regulation, it has been suggested that individuals exposed to ACE may benefit from using nicotine for regulation of mood [69]. Thus, for those individuals, attempts to quit may remove nicotine as their pharmacological coping device for the negative emotional, neurobiological, and social effects of ACE [68].

Overall, there are profound implications from the findings that childhood difficulties are associated with increased odds of unhealthy lifestyle behaviors and increased risk of living with obesity in adulthood. Screening and detection of childhood difficulties should be improved in order to provide services and support that may reduce the risk of negative health consequences. In addition, the development of tailored obesity prevention and treatment programs for individuals who have a difficult childhood should be considered.

Moreover, a recent study demonstrating significant associations between experience of childhood abuse, low self-esteem, and negative experiences interacting with health care providers has concluded that health personnel should receive training to ensure open and nonjudgmental visits with obese patients and consider the role of trauma survivorship issues in patients’ development of obesity and health care experiences [70].

### Strength and Limitations

Results from the present study should be interpreted with caution due to certain methodological limitations, including the cross-sectional design, which cannot be used to infer causality [71]. Another weakness of the present study is the limited participation rate, which must nevertheless be seen as acceptable among similar national and international studies among adults. Although a large sample of adults was randomly drawn from a general population, our study included a higher proportion of higher-educated adults compared the total adult population in Norway (48% versus 35%) [72]. However, as low educational level is both associated with increased prevalence of unhealthy lifestyle behaviors [73,74] and ACEs [28], this selection bias could lead to an underestimating of the association but most likely not the direction of our results. Further, we must acknowledge the possibility of bias due to misreporting of childhood experiences, which may be explained by recall bias or due to suppression of traumatic memories [10]. However, results from previous studies comparing retrospective and prospective data on ACEs have not shown evidence of recall bias [75,76,77].

Information on childhood difficulties were assessed using a single question reflecting a subjective, global evaluation of childhood, which previously has been proven useful for identification of a graded association between having a difficult childhood and multimorbidity, individual disease burden, and biological perturbations [9]. Whereas most other studies have focused on specified types of adverse events in childhood [78], a strength of our study is that the focus on childhood in a global perspective addresses the respondent’s personal appraisal of what might be described as the overall balance between adverse and supporting and resilience factors in childhood [79]. Moreover, studies have reported that not only the number of adversities but also how individuals rate the impact of adverse experiences were of importance [80] and that short ACE-instruments that are more reflective have good discriminant validity [31]. In general, application of single questions to measure childhood difficulties may be important to increase the inclusion of this subject in population-based studies.

In addition, a strength of the present study was use of a validated tool to measure alcohol risk drinking (AUDIT-C), and previous studies have shown that this instrument is reliable and a valid short form of the original version [81]. For other outcome variables, we used cut-off values that have previously been used in similar studies among adults and therefore allow comparison of results.

## 5. Conclusions

Results from the present cross-sectional study confirmed an association between self-perceived childhood difficulties and increased likelihood of living with obesity and being involved in a range of unhealthy lifestyle behaviors in adulthood. Future longitudinal studies are needed to confirm a possible causal relationship between childhood difficulties and various lifestyle behaviors and adult morbidity, which may help practitioners select appropriate intervention methods and strategies to improve health and quality of life among individuals with ACEs. In order to disseminate results from the present study in a way that may positively affect people’s life, this study will be presented to regional and national public health authorities, health personnel, and other public health officials.

## Figures and Tables

**Table 1:** Questions, response alternatives, and variable definitions.

Questions	Response Alternatives	Variable Definitions
**Childhood Difficulties**		
When you think about your childhood, would you describe it as:	Very good, good, average, difficult, very difficult	Childhood difficulties (difficult or very difficult) vs. no adverse childhood experiences (ref.)
**BMI**		
How tall are you in your bare feet? (in cm)		BMI ≥ 30 (obesity) or BMI < 30 (ref.)
How much do you weigh without clothes or shoes? (in kg) If pregnant, state pre-pregnancy weight.		
**Diet**		
How often do you usually consume sugar-sweetened soft drinks or beverages?	Rarely/never, 1–3 times/month, one time/week, 2–3 times/week, 4–6 times/week, daily	High consumption of SSBs (≥4 times a week) vs. low consumption of SSBs (ref.)
How often do you usually eat fruit or berries (not including juice)?		Low consumption of fruit and berries (<once a day) vs. high consumption of fruit and berries (ref.)
How often do you usually eat vegetables (including salads)?		Low consumption of vegetables (<once a day) vs. high consumption of vegetables (ref.)
How often do you usually eat fish (as a sandwich spread or for a meal)?		Low consumption of fish (<once a week) vs. high (ref.)
**Leisure-time physical activity**		
How often do you exercise? (average)	Never, <once a week, 2–3 times a week, 4–5 times a week, ≥6 times/week I take it easy without being breathless or sweaty (low intensity); I push myself until I breathe faster and am sweating (moderate intensity); I practically exhaust myself (high intensity) < 15 min, 15–29 min, 30–60 min, >60 min	Adherence to physical activity recommendations (75 min/week or more with high intensity or 150 min/week or more with moderate intensity) vs. not adhering to physical activity recommendations (ref.)

How hard do you exercise? (average)		
For how long do you exercise each time? (average)		
**Tobacco: smoke and the use of smokeless tobacco**		
How often do you smoke? Include both filter cigarettes and rolling tobacco.	Daily, occasionally, not now but previously daily, not now but previously occasionally, or never smoked/used smokeless tobacco	Current use (daily or occasionally) vs. no current use (ref)
How often do you use smokeless tobacco?		
**Alcohol**		
Have you ever consumed alcohol?	Yes, no	A total sum score between 0 and 12 points was calculated. Excessive alcohol use (≥5 men, ≥4 women) vs. no-risk drinking (ref.)
During the last 12 months, how often have you consumed alcohol?	Never, ≤once/month, 2–4 times/month, 2–3 times/week, ≥4 times/week	
How many units of alcohol do you drink on a “typical” day when you drink alcohol?	1–2, 3–4, 5–6, 7–9, ≥10	
How often do you drink six or more units of alcohol in a single session?	Never, <once/month, every month, every week, daily/nearly daily basis	
**Education**		
What is your highest completed level of education?	Primary school (up to 10 years of education), secondary school/high school (up to 13 years of education), university or college (up to 16 years of education), university or college (16 years of education or more)	University/college and secondary school vs. primary school (ref)
**Age**		
Retrieved from the Central Population Register	≥18 years old	Continuous variable (low, ref.)

**Table 2:** Characteristics of participants according to self-perceived childhood difficulties.

Childhood Difficulties						
	Very Good	Good	Average	Difficult	Very Difficult	*p*-Value *
***Number of participants, n (%)***	13,938 (49.8)	8252 (29.5)	3460 (12.4)	1657 (5.9)	689 (2.5)	Na
***Gender, n (%)***						
Female	7163 (51.4)	4241 (51.4)	1992 (57.6)	1063 (64.2)	442 (64.2)	<0.001
Male	6773 (48.6)	4011 (48.6)	1468 (42.4)	594 (35.8)	247 (35.8)	
***Age, mean (SD)***	47.8 (±16.7)	47.0 (±16.0)	45.8 (±16.0)	43.6 (±14.8)	39.8 (±13.7)	<0.001
***Educational level, n (%)***						
Primary school	1477 (10.6)	919 (11.2)	479 (13.9)	267 (16.2)	187 (27.4)	<0.001
Secondary school	5452 (39.2)	3258 (39.6)	1382 (40.1)	686 (41.5)	299 (43.8)	
University/college	6970 (50.1)	4041 (49.2)	1583 (46.0)	700 (42.3)	197 (28.8)	
***BMI, n (%)***						
Underweight	118 (0.9)	101 (1.3)	57 (1.7)	29 (1.8)	19 (2.8)	<0.001
Normal weight	5924 (43.3)	3481 (43.2)	1418 (42.0)	662 (41.0)	276 (41.3)	
Overweight	5361 (39.2)	3021 (37.5)	1226 (36.3)	580 (36.0)	219 (32.7)	
Obesity, class I	1712 (12.5)	1082 (13.4)	473 (14.0)	249 (15.4)	102 (15.2)	
Obesity, class II	554 (4.1)	380 (4.7)	200 (5.9)	93 (5.8)	53 (7.9)	
***Food and beverages, n (%)***						
**Fruit and berries**						
Low (<once a day)	9612 (69.1)	5897 (71.6)	2551 (72.7)	1232 (74.4)	534 (77.6)	<0.001
**Vegetables**						
Low (<once a day)	8739 (62.8)	5313 (64.5)	2211 (64.0)	1065 (64.3)	453 (65.9)	0.071
**Fish**						
Low (<once a week)	2941 (21.1)	2035 (24.7)	985 (28.5)	548 (33.1)	256 (37.3)	<0.001
**Sugar-sweetened beverages**						
High (≥4 times a week)	3113 (22.4)	1914 (23.2)	834 (24.1)	401 (24.2)	200 (29.1)	<0.001
***Physical activity (PA), n (%)***						
Low level of PA	8739 (63.1)	5368 (65.5)	2226 (65.7)	1105 (67.2)	469 (68.3)	<0.001
***Alcohol, n (%)***						
Excessive alcohol use	5086 (36.6)	2997 (36.4)	1268 (36.8)	648 (39.3)	255 (37.1)	0.280
***Smoking, n (%)***						
Current smoker	1855 (13.3)	1193 (14.5)	612 (17.7)	378 (22.8)	196 (28.4)	<0.001
***Smokeless tobacco, n (%)***						
Current user	1777 (12.8)	1126 (13.7)	539 (15.6)	254 (15.4)	148 (21.5)	<0.001

Standard deviation (SD) and percentages (%) within brackets as appropriate. * *p*-trend calculated with chi-square test (categorical variables) and one-way ANOVA (continuous variables) indicate childhood difficulties (ranging from very good to very difficult) according to participant characteristics.

**Table 3:** Adjusted odds ratio (OR) and 95% confidence interval (CI) for being overweight or obese and having unhealthy dietary habits and low physical activity level in relation to self-perceived childhood difficulties.

Variables		Obesity	Fruit and Berries, Low	Vegetables, Low	Fish, Low	Sugar-Sweetened Beverages, High	Physical Activity, Low
		OR (CI 95%)	OR (CI 95%)	OR (CI 95%)	OR (CI 95%)	OR (CI 95%)	OR (CI 95%)
**Exposure** **Variable**	**Childhood difficulties**						
	No	1.0	1.0	1.0	1.0	1.0	1.0
	Yes	1.29 (1.16–1.44) ***	1.21 (1.09–1.34) ***	1.03 (0.94–1.13)	1.43 (1.30–1.57) ***	1.30 (1.14–1.48) ***	1.10 (1.01–1.21) *
**Control** **Variables**	**Gender**						
	Women	1.0	1.0	1.0	1.0	1.0	1.0
	Men	1.18 (1.10–1.25) ***	2.37 (2.24–2.51) ***	2.40 (2.27–2.52) ***	1.07 (1.01–1.14) *	1.98 (1.82–2.15) ***	0.97 (0.93–1.02)
	**Age**						
	Younger Adults	1.0	1.0	1.0	1.0	1.0	1.0
	Older Adults	1.00 (1.00–1.01) ***	0.97 (0.97–0.97) ***	0.98 (0.98–0.99) ***	0.96 (0.96–0.97) ***	0.97 (0.96–0.97) ***	1.00 (1.00–1.00)
	**Education**						
	Primary School	1.0	1.0	1.0	1.0	1.0	1.0
	Secondary School	0.79 (0.72–0.87) ***	0.85 (0.78–0.94) **	0.85 (0.77–0.92) ***	0.76 (0.70–0.84) ***	0.70 (0.63–0.79) ***	0.81 (0.75–0.88) ***
	University/College	0.56 (0.51–0.62) ***	0.61 (0.56–0.67) ***	0.54 (0.50–0.59) ***	0.50 (0.45–0.54) ***	0.35 (0.31–0.40) ***	0.66 (0.61–0.72) ***

Indicates a statistic significance *p* < 0.05 *, *p* < 0.01 **, *p* < 0.001 ***.

**Table 4:** Adjusted odds ratio (OR) and 95% confidence interval (CI) for substance use in relation to self-perceived childhood difficulties.

Title		Alcohol Excessive Use	Smoking Current Use	Smokeless Tobacco Current Use
		OR (CI 95%)	OR (CI 95%)	OR (CI 95%)
**Exposure Variable**	**Childhood difficulties**			
	No	1.0	1.0	1.0
	Yes	0.96 (0.89–1.05)	1.78 (1.61–1.97) ***	1.20 (1.07–1.36) ***
**Control Variables**	**Gender**			
	Women	1.0	1.0	1.0
	Men	1.04 (0.99–1.10)	1.07 (1.00–1.15) *	2.59 (2.41–2.80) ***
	**Age**			
	Younger Adults	1.0	1.0	1.0
	Older Adults	0.97 (0.97–0.98) ***	1.00 (1.00–1.00) ***	0.95 (0.95–0.95) ***
	**Education**			
	Primary School	1.0	1.0	1.0
	Secondary School	1.06 (0.97–1.15)	0.65 (0.59–0.72) ***	0.84 (0.74–0.94)
	University/College	0.89 (0.82–0.97) **	0.35 (0.32–0.39) ***	0.75 (0.66–0.84)

Indicates a statistic significance *p* < 0.05 *, *p* < 0.01 **, *p* < 0.001 ***.

## Data Availability

Restrictions apply to the availability of these data. Data was obtained from Norwegian Institute of Public Health (NIPH) and are available at https://helsedata.no/en (accessed on 27 April 2021) with the permission of NIPH.
